# Markers for the angiogenic potential of fat grafts

**DOI:** 10.1007/s00508-025-02532-8

**Published:** 2025-04-15

**Authors:** Maryana Teufelsbauer, Sandra Stickler, Marie-Therese Eggerstorfer, Dennis C. Hammond, Clemens Lang, Gerhard Hamilton

**Affiliations:** 1 Department of Plastic, Reconstructive and Aesthetic Surgery, Medical University of Vienna, 1090 Vienna, Austria; 2 Institute of Pharmacology, Medical University of Vienna, Waehringerstraße 13A, 1090 Vienna, Austria; 3 Center for Breast and Body Contouring, 49546 Grand Rapids, MI USA; 4 Department of Trauma Surgery, Hospital Donaustadt, 1220 Vienna, Austria

**Keywords:** Liposuction, Adipose-derived stromal cells, Adipokines, Angiogenesis, VEGF

## Abstract

**Background:**

Fat grafting is widely utilized in reconstructive and esthetic plastic surgery, typically with minimal complications. Nevertheless, the occurrence of fat necrosis is dependent on the technique used for fat extraction, tissue processing and the volume of the graft. The longevity of the graft critically depends on the presence of adipose-derived stromal cells (ADSC) and their promotion of a reconstituted vascular supply.

**Objective:**

This study seeks to determine whether there are differences in 13 angiogenesis-related adipokines based on their grouping by vascular endothelial growth factor (VEGF) expression levels.

**Methods:**

The expression of 14 adipokines related to angiogenesis in 12 cultured ADSCs was evaluated using Human Adipokine Profiler kits, which simultaneously detect 58 mediators. Adipokines of the high and low VEGF expression groups were evaluated for their expression of the remaining 13 angiogenic proteins.

**Results:**

We were able to show that there are significant differences in VEGF^low^ and VEGF^high^ ADSCs regarding fibroblast growth factor 19 (*p* = 0.043) and insulin like growth factor binding protein 3 (*p* = 0.028). Furthermore, ADSCs with differentially highly expressed VEGF show a different pattern in the amount of protein levels regarding the 13 other adipokines observed.

**Conclusion:**

The VEGF has been described as a key angiogenic factor in fat grafts that may be linked to successful grafting; however, two of the fat samples analyzed exhibited high expression of VEGF but lacked significant co-expression of a range of other angiogenic factors. Thus, the assessment of the expression of predisposing mediators for graft angiogenesis for wound healing or contouring should include further angiogenesis promoters aside VEGF as parameters.

## Introduction

Although autologous fat transplantation (AFT) in patients shows excellent biocompatibility and simple applicability, the relatively low retention rate caused by fat necrosis is still a challenge [1, 2]. Lipotransfer or AFT promotes wound healing, scar reduction and can be used for contouring defects and breast reconstruction [3]. Rapid and efficient angiogenesis within grafts is essential for supplying oxygen to secure the survival of adipocytes. The vasculature likewise provides a niche for interaction between adipose and vascular progenitor cells, thus enhancing angiogenesis and adipogenesis in grafts. Various methods, such as enriching grafts with diverse pro-angiogenic cells or utilizing cell-free approaches, have been employed to enhance angiogenesis.

Suction-harvested adipose tissue lacks its natural vascular network essential for providing oxygen and nutrients. This deficiency may cause deleterious volume reduction, fibrosis and the development of oil cysts due to fat necrosis [4–6]. Thus, early revascularization is a crucial prerequisite to achieve high survival rates after AFT [7]. The regenerative potential of grafts relies on the rapid ingrowth of capillaries that provide oxygen to prevent a state of severe ischemia and hypoxia [8–10]. Most adipocytes in the graft begin to die within 24 h but adipose-derived stroma cells (ADSCs) remain viable for up to 72 h, exhibiting at least limited resistance to hypoxia. Notably, the viability and yield of ADSCs can vary depending on the donor area, as shown by Tsekouras et al., with the lower abdomen and flanks providing the highest ADSC yield and viability, making them the preferred sources for transplantation [11]. It takes 3–7 days for the peripheral vessels to invade the outer borders of the grafts [12]. Therefore, smaller grafts show better survival rates in general compared with larger grafts due to the more favored surface-to-volume ratio [13, 14]. Co-culturing endothelial progenitor cells (EPCs) with ADSCs enhances tube formation potential and promotes angiogenesis effectors, such as angiopoietin-1 receptor (Tie2) and vascular endothelial growth factor (VEGF). The combination of ADSCs enhances the functional capacity of EPCs in the fat graft instead of direct ADSC/EPC differentiation [15]. Supporting AFTs with diverse cell types that hold significant angiogenic activity, such as ADSCs, is a highly effective approach to enhance the neovascularization of transplanted adipose tissue.

Fat tissue is a rich source of pluripotent ADSCs that can differentiate into a range of cell types, such as adipocytes, osteoblasts, endothelial cells, chondrocytes and others [16, 17]. Adipose-derived stroma cells express the surface markers CD73, CD90, CD105, and STRO‑1 and a high replication capacity and as well as the ability to secrete a multitude of pro-angiogenic growth factors [18, 19]. The utilization of exogenously added ADSCs in fat grafting has been termed cell-assisted lipotransfer (CAL) [20]. Vascular endothelial growth factor serves as an excellent target for enhancement of paracrine function of ADSCs by promotion of vascular networks within grafts. The VEGF induces proliferation, survival, sprouting, migration and tube formation of endothelial cells (ECs) [21–23]. Furthermore, 3D-cultured ADSCs demonstrate increased proliferation potential and reduced senescence compared with 2D culture methods. Co-culturing 3D-cultured ADSCs with human umbilical vein endothelial cells (HUVECs) enhances their neovascularization capabilities. We have demonstrated in two previous publications that adipokines and concentrations of VEGF are highly variable in potential fat grafts obtained by liposuction [24, 25].

This study aims to investigate differences in expression levels of angiopoietins fibroblast growth factor 2 (FGF basic), fibroblast growth factor 19 (FGF-19), hepatocyte growth factor (HGF), intercellular adhesion molecule 1 (ICAM-I/CD54), insulin like growth factor binding protein 2 (IGFBP-2), insulin like growth factor binding protein 3 (IGFBP‑3), tissue inhibitor of metalloproteinases 1 (TIMP‑1), tissue inhibitor of metalloproteinases 3 (TIMP‑3) and tumor necrosis factor (TNF-alpha) in relation to low and high VEGF expression levels of liposuction donors. These adipokines were selected due to their well-established roles in angiogenesis. Among them, angiopoietin‑1, angiopoietin-like 2, angiopoietin-like 3, FGF basic, FGF-19, HGF, IGFBP‑2 and IGFBP‑3 have shown to promote angiogenesis [26–32]. In contrast, ICAM-I/CD54, TIMP‑1 and TIMP‑3 are recognized for their anti-angiogenic effects [33–36]. Additionally, angiopoietin‑2 and TNF-alpha exhibit dual roles, acting either as pro-angiogenic or anti-angiogenic regulators depending on the cellular context [37–39]. Understanding the expression patterns of these factors in relation to VEGF levels may provide valuable insights into the regulatory mechanisms of angiogenesis and its potential therapeutic implications. The highest success rates of AFT are associated with the grafts’ early angiogenic capacity.

## Patients, material and methods

Lower abdominal liposuction was conducted on 12 female patients using a 0.5 mm cannula and 50 ml aliquots of the samples were transferred for experimental studies. Recovery of fat and isolation of ADSCs were performed with written informed consent of the patients according to the Ethics Approval 366/2003 of the Ethics Committee of the Medical University of Vienna (Vienna, Austria). All procedures followed were in accordance with the ethical standards of the responsible committee on human experimentation (institutional and national) and with the Helsinki Declaration of 1975, as revised in 2008. The extracted fat particles were first washed with tissue culture medium consisting of RPMI-1640 medium (Sigma-Aldrich, St. Louis, MO, USA) supplemented with 30% fetal bovine serum (Eximus, Catus Biotech, Tutzing, Germany) and antibiotics (Sigma-Aldrich) [40]. The washed fat particles were incubated under cell culture conditions at 37 °C and 5% CO_2_ in 75 cm^2^ tissue culture flasks (Greiner Bio-One GmbH, Kremsmuenster, Austria) until outgrowth of ADSCs. The ADSCs were subsequently expanded, while any remaining fat tissue was discarded. The ADSC phenotype of the cells was confirmed by flow cytometry using a Cytomics FC500 FACS (Beckman Coulter Germany GmbH, Krefeld, Germany) and positive reactivity with monoclonal antibodies directed against CD105, CD90 and CD73 and lacking reactivity with the hematopoietic marker CD34 [41].

Conditioned supernatants of the ADSC cultures were analyzed by Human Adipokine Arrays (ARY024, R&D Systems, Minneapolis, MN, USA) that simultaneously detects 58 obesity-related adipokines. The experiments were carried out in accordance with the instructions provided by the manufacturer and all experiments were performed in duplicate. Individual pixel intensities of the samples were calibrated using the reference spots and pixel values were normalized to ensure comparability. The arrays were analyzed using Quickspot (Ideal Eyes System, Bountiful, UT, USA) and Origin 9.1 software (OriginLab, Northampton, MA, USA). Due to their relevance regarding angiogenesis 14 of the 58 adipokines were chosen for further analysis. In accordance with results of our recent publications VEGF expression was used as the distinguishing criterion for the selection of two groups [41]. The threshold distinguishing low and high VEGF expression levels was determined using K-Means clustering, which identified a cutoff value of 10,706. Patients were then grouped based on this threshold [42]. Furthermore, a pathway analysis comprising these 14 proteins was conducted using the Reactome databank (www.reactome.org) [43]. T‑tests were used to determine statistical significance, with a *p*-value of less than 0.05 considered significant.

## Results

**Fig. 1:**
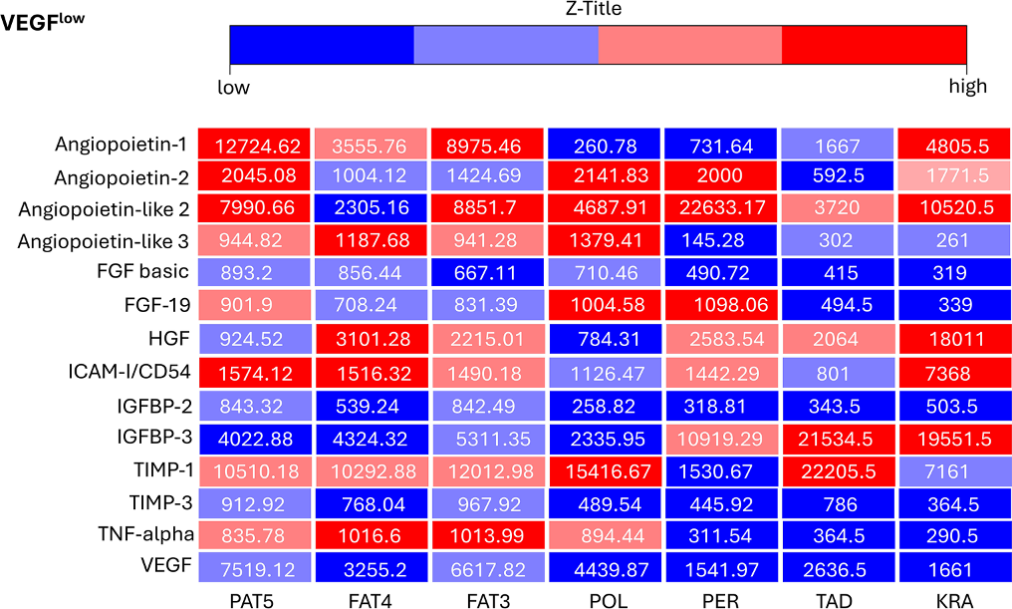
This heatmap shows the relative levels (in arbitrary pixel values) of 14 proteins involved in angiogenesis, as evaluated by two independent Human Adipokine Arrays (Catalog #ARY024, R&D Systems, Minneapolis, MN, USA). These arrays were performed on seven vascular endothelial growth factor^low^ adipose-derived stroma cell samples: PAT5, FAT4, FAT3, POL, PER, TAD, KRA. Standard deviation of all measurements was below 12%. *FGF basic* fibroblast growth factor 2, *FGF-19* fibroblast growth factor 19, *HGF* hepatocyte growth factor, *ICAM-I/CD54* intercellular adhesion molecule 1, *IGFBP‑2* insulin like growth factor binding protein 2, *IGFBP‑3* insulin like growth factor binding protein 3, *TIMP‑1* tissue inhibitor of metalloproteinases 1, *TIMP‑3* tissue inhibitor of metalloproteinases 3, *TNF-alpha* tumor necrosis factor, *VEGF* vascular endothelial growth factor

**Fig. 2:**
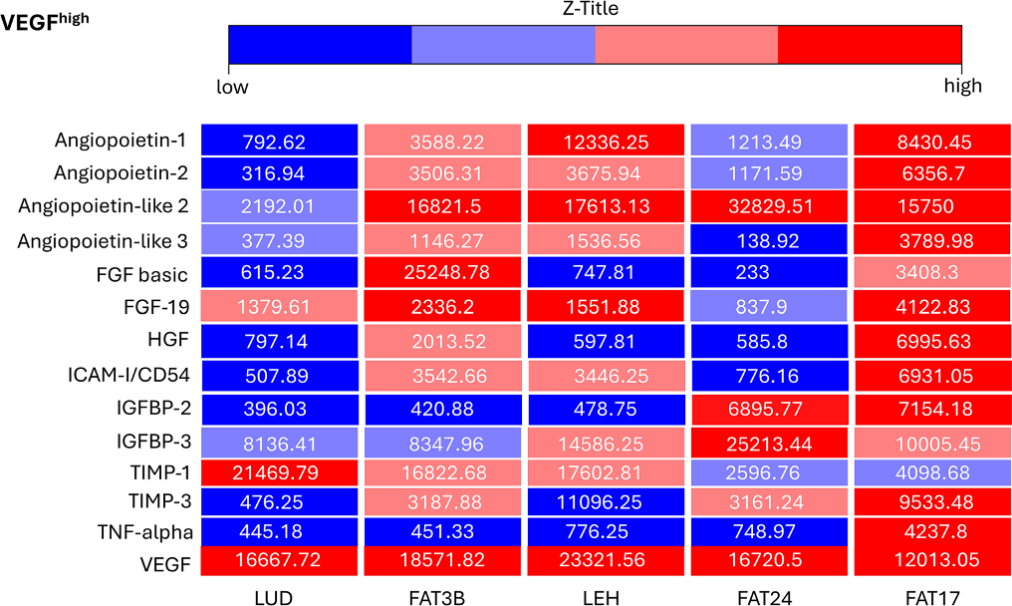
This heatmap shows the relative levels (in arbitrary pixel values) of 14 proteins involved in angiogenesis, as evaluated by two independent Human Adipokine Arrays (Catalog #ARY024, R&D Systems, Minneapolis, MN, USA). These arrays were performed on five vascular endothelial growth factor^high^ adipose-derived stroma cell samples: LUD, FAT3B, LEH, FAT24, FAT17. Standard deviation of all measurements was below 12%. *FGF basic* fibroblast growth factor 2, *FGF-19* fibroblast growth factor 19, *HGF* hepatocyte growth factor,* ICAM-I/CD54* intercellular adhesion molecule 1, *IGFBP‑2* insulin like growth factor binding protein 2, *IGFBP‑3* insulin like growth factor binding protein 3, *TIMP‑1* tissue inhibitor of metalloproteinases 1, *TIMP‑3* tissue inhibitor of metalloproteinases 3, *TNF-alpha* tumor necrosis factor, *VEGF *vascular endothelial growth factor

**Fig. 3:**
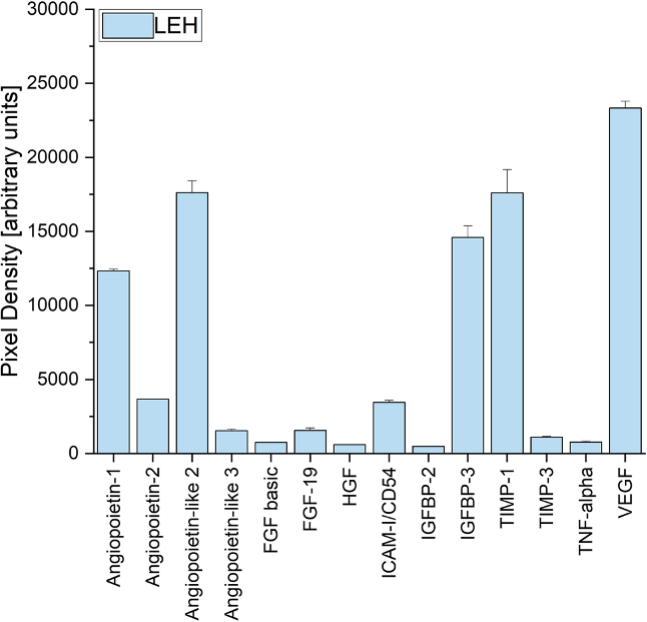
The levels of 14 angiogenesis-related adipokines of vascular endothelial growth factor^high^ (VEGF^high^) adipose-derived stroma cells of patient LEH. This data was calculated via two independent Human Adipokine Arrays (Catalog #ARY024, R&D Systems, Minneapolis, MN, USA). *FGF* *basic* fibroblast growth factor 2, *FGF-19* fibroblast growth factor 19, *HGF* hepatocyte growth factor, *ICAM-I/CD54* intercellular adhesion molecule 1, *IGFBP‑2* insulin like growth factor binding protein 2, *IGFBP‑3* insulin like growth factor binding protein 3, *TIMP‑1* TIMP metallopeptidase inhibitor 1, *TIMP‑3* TIMP metallopeptidase inhibitor 3, *TNF-alpha* tumor necrosis factor, *VEGF* vascular endothelial growth factor

The most relevant angiogenic factors were assessed for ADSCs with high (VEGF^high^ *n* = 5) and low VEGF (VEGF^low^ *n* = 7) expression (Figs. 1 and 2). These adipokines are angiopoietin‑1, angiopoietin‑2, angiopoietin-like 2, angiopoietin-like 3, FGF basic, FGF-19, HGF, ICAM-I/CD54, IGFBP-2, IGFBP-3, TIMP-1, TIMP-3 and TNF-alpha and VEGF. Heatmaps were chosen to depict differences in the levels of these 14 adipokines in ADSCs with either VEGF^low^ or VEGF^high^ (Figs. 1 and 2). While there were several observable differences between VEGF^low^ and VEGF^high^ ADSCs, only FGF-19 (*p* = 0.043) and IGFBP‑3 (*p* = 0.028) showed statistically significant differences. The Human Adipokine Array shown provides an example of a single array as demonstrated by the VEGF^high^ conditioned supernatant of ADSCs of patient LEH (Fig. 3).

The reactome analysis of 14 proteins involved in angiogenesis revealed several significantly expressed pathways. Interleukin‑4 (IL-4) and interleukin-13 (IL-13) signaling exhibited the highest *p*-value, followed by factor receptor 4 (FGFR4) ligand binding and activation; nuclear receptor subfamily 1 group H members 2 and 3 (NR1H2 and NR1H3) regulation of gene expression linked to lipogenesis, phospholipase C‑mediated cascade FGFR4, POU class 5 homeobox 1 (POU5F1), SRY-box transcription factor 2 (SOX2) and nanog homeobox (NANOG), activate genes related to proliferation; phosphatidylinositol‑4,5‑bisphosphate 3‑kinase catalytic (PI-3K) cascade: FGFR4; regulation of insulin-like growth factor (IGF) transport and uptake by insulin-like growth factor binding proteins (IGFBPs); SHC adaptor protein 1 (SHC)-mediated cascade: FGFR4; transcriptional regulation of pluripotent stem cells and insulin receptor substrate (IRS)-mediated signaling (Table 1).

**Table 1:** The same 14 angiogenic proteins were subjected to a cellular pathway analysis: Pathways are listed according to the components/entities of specific cascades found (ratio of number of entities/total number of pathway members)

	Entities			
	Found	Ratio	*p*-value	FDR*
IL‑4 and IL-13 signaling	10/211	0.013	3.01e–14	4.18e–12
IL-10 signaling	6/86	0.005	9.55e–10	6.59e–08
FGFR4 ligand binding and activation	2/17	0.001	2.18e–04	0.005
NR1H2 and NR1H3 regulate gene expression linked to lipogenesis	2/17	0.001	2.18e–04	0.005
Phospholipase C‑mediated cascade; FGFR4	2/19	0.001	2.72e–04	0.005
POU5F1, SOX2, NANOG activate genes related to proliferation	2/21	0.001	3.32e–04	0.005
PI-3K cascade: FGFR4	2/25	0.002	4.69e–04	0.006
Regulation of IGF transport and uptake by IGFBPs	3/127	0.008	5.37e–04	0.006
SHC-mediated cascade: FGFR4	2/27	0.002	5.46e–04	0.006
Transcriptional regulation of pluripotent stem cells	2/45	0.003	0.001	0.012
IRS-mediated signalling	2/65	0.004	0.003	0.015

The false discovery rate (FDR) ranged from 4.18e–12 to 0.015, from pathways with highest to lowest *p*-values

*IL‑4* interleukin 4, *IL-13* interleukin 13, *IL-10* interleukin 10, *FGFR4* fibroblast growth factor receptor 4, *NRH1H2* nuclear receptor subfamily 1 group H member 2, *NR1H3* nuclear receptor subfamily 1 group H member 3, *TP53* tumor protein P53, *POU5F1* POU class 5 homeobox 1, *SOX2* SRY-box transcription factor 2, *NANOG* Nanog homeobox, *PI-3K* phosphatidylinositol‑4,5‑bisphosphate 3‑kinase catalytic, *IGF* insulin-like growth factor, *IGFBPs* insulin-like growth factor binding proteins, *SHC* SHC adaptor protein 1, *IRS* insulin receptor substrate

## Discussion

Liposuction and autologous fat transfer (AFT) are increasingly being used in plastic and reconstructive surgery to promote wound healing and to fill defects as well as for body contouring. Furthermore, AFT is an emerging therapeutic option for the treatment of wounds that are not suitable for grafting. The regenerative potential of autologous fat lies in the ADSCs, which are capable of differentiating into multiple cell lineages and secreting various adipokines. Human fat products could accelerate the healing rate, shorten recovery and achieve more complete healing compared to conventional treatment. Histological findings indicated that fat extracts promote epithelialization, collagen deposition and vascularization, thereby facilitating tissue regeneration and reducing inflammatory reactions [44]. The surgical retrieval of fat is usually without problems but the successful yield of the transferred tissue is variable and may end in fat necrosis and failure of the graft in up to 30% of the cases [45]. The most critical point for the survival of the graft is the early and efficient restoration of the vessel supply and the provision of oxygen and nutrients. Of course, smaller fat particles survive better than larger ones and repeated grafting of smaller samples results in a better outcome. Furthermore, the grafts may be enriched with specific cells or mediators that exert an angiogenic effect and support the vessel supply. This may be in the form of additional ADSCs (cell-supported ATF) or in the coadministration of angiogenic factors such as the most active agent VEGF.

**Fig. 4:**
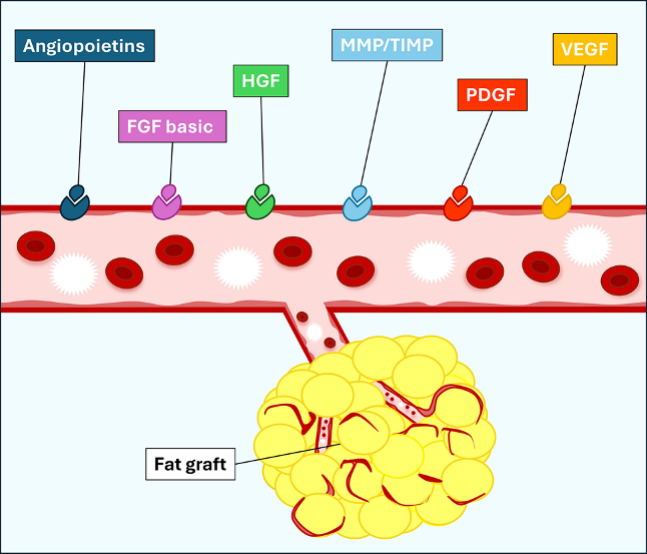
Diagram of the mechanisms following a fat graft showing the roles of various factors. *FGF basic* fibroblast growth factor 2, *HGF *hepatocyte growth factor, *MMP/TIMP* matrix metalloproteinase/tissue inhibitor of metalloproteinases, *PDGF* platelet-derived growth factor, *VEGF* vascular endothelial growth factor

Obesity is usually accompanied by inflammation of fat tissue, with a prominent role of visceral fat [46]. Proinflammatory signaling of adipocytes and other cells causes the resident immune system to release increased amounts of proinflammatory and other mediators resulting in enhanced tissue-protective responses. Angiogenic factors like angiopoietins, FGF basic, HGF, matrix metalloproteinase/tissue inhibitor of metalloproteinases (MMP/TIMP), platelet derived growth factor (PDGF) and VEGF play crucial roles in the survival and integration of fat grafts. These factors enhance the formation of new blood vessels, which is vital for the viability of transplanted fat tissue. Vascular endothelial growth factor stimulates endothelial cells, promoting new capillary growth towards the graft, while angiopoietins stabilize the new vessels. Tissue inhibitor of metalloproteinases and TIMPs remodel the extracellular matrix, allowing new vessels to integrate effectively. The platelet derived growth factor attracts and activates cells necessary for repair and regeneration, further supporting graft incorporation. Hepatocyte growth factor contributes by enhancing angiogenesis and supporting the proliferation and migration of cells. Fibroblast growth factor 2 promotes the formation of new blood vessels and assists in tissue repair by stimulating the proliferation and survival of various cell types within the transplanted fat. Overall, these factors ensure that the fat graft receives sufficient blood supply, which is crucial for its successful incorporation and longevity (Fig. 4). With chronic overnutrition of adipose tissues, these protective actions are insufficient and death of adipocytes as well as senescence of several tissue cell types is seen.

Adipose tissues located in subcutaneous areas (SAT) and visceral adipose tissue (VAT) of the abdominal cavity show important differences [47]. Compared with SAT the VAT is more cellular, vascular and hosts a larger number of inflammatory cells, fewer preadipocytes but more large adipocytes. Remodeling in visceral fat during obesity results in hypertrophy and death of adipocytes, hypoxia, inflammation, and fibrosis [48]. Furthermore, visceral fat secretes more fatty acids and proinflammatory and profibrotic cytokines [49]. Finally, almost half of the adipose tissue secretome comprises factors functional in angiogenesis [50]. Metabolically active adipokines including leptin and adiponectin are secreted in higher amounts from SAT whereas proinflammatory adipokines such as retinol binding protein 4 (RBP4), TNF-alpha, monocyte chemotactic and activating factor (MCP‑1), interleukin 8 (IL‑8), and interleukin 6 (IL‑6) are increased in VAT [51]. While leptin and adiponectin are secreted by adipocytes, other factors including resistin, visfatin, TNF-alpha, IL‑6, and MCP‑1 are principally secreted by macrophages; however, several reports of anti-angiogenic treatment resulted not only in lower adiposity but also in improved metabolic parameters [52].

In an experimental animal model, the delivery of IL-4-releasing microparticles did not affect the density or size of blood vessels as measured by immunohistochemical analysis but it did increase the perfused tissue volume as measured by 3D microcomputed tomography [53].

Pretesting of the ADSCs of a fat graft would be of special interest for allogeneic grafting. The ADSCs exhibit low immunogenicity and can be processed, collected and stored for transfer to patients not related to the donor. The safety and therapeutic potential of allogeneic adipose-derived stem cell spray transplantation in ischemic cardiomyopathy was published [54]. The safety and efficacy of allogenic ADSCs in human diabetic foot ulcer treatment was recently evaluated. The investigators discovered that the time to 50% reduction of wound size was significantly shorter in patients who received ADSCs. Complete healing was achieved at the end of the study in seven patients treated with ADSCs vs. one treated without ADSCs [55].

In this study, we aimed to identify expression patterns of 14 angiogenesis-related genes in ADSCs within a VEGF^low^ and a VEGF^high^ group, correlating with their pro-angiogenic or anti-angiogenic effects. Understanding how ADSCs interact with VEGF is essential as VEGF is a key mediator of angiogenesis and plays a crucial role in the survival and function of transplanted fat grafts. Angiopoietin‑1 functions as a ligand for the TIE2 receptor, stabilizing blood vessels and protecting them against VEGF-induced plasma leakage [26, 56]. Liu et al. demonstrated that angiopoietin-like 2 enhances endothelial cell migration and angiogenesis by stimulating VEGF‑A production in human lung cancer cells, facilitating lymphangiogenesis through the integrin α5β1, p38 and NF-κB pathways [57]. Similarly, Zhong et al. found that angiopoietin-like 3 promotes cell proliferation, migration, and angiogenesis by binding to integrin αvβ3, activating downstream signaling pathways such as PI3K/Threonine kinase 1 (AKT), leading to increased VEGF expression [58]. Further supporting VEGF-driven angiogenesis, Seghezzi et al. were the first to demonstrate that FGF basic upregulates VEGF expression, leading to increased endothelial cell proliferation [59]. In addition, it was found that FGF-19 enhances angiogenesis in nasopharyngeal carcinoma by preventing tripartite motif containing 21 (TRIM21)-mediated Annexin II degradation, thereby increasing VEGF expression [60]. Xin et al. further established that HGF stimulates angiogenesis by promoting VEGF expression and driving endothelial cell proliferation and migration [30]. Furthermore, IGFBP‑2 directly activates the VEGF gene promoter, upregulating VEGF expression and facilitating angiogenesis in neuroblastoma cells [61]. Granata et al. showed that IGFBP‑3 influences angiogenesis by enhancing tube formation and upregulating VEGF-related gene expression in endothelial cells [32]. Despite the expectation that elevated expression levels of angiopoietin‑1, angiopoietin-like 2, angiopoietin-like 3, FGF basic, FGF-19, HGF, IGFBP‑2 and 3 would correlate with high VEGF levels, no such association was observed (Fig. 2).

Moreover, Griffioen et al. demonstrated that ICAM-I/CD54 plays a role in inflammation and can regulate angiogenesis by interacting with VEGF signaling [34]. Qi et al. demonstrated that TIMP‑1 enhances VEGF-induced retinal neovascularization, highlighting a tissue-specific role in angiogenesis [36]. Yamada et al. further found that TIMP‑1 enhances VEGF-induced retinal neovascularization, with overexpression increasing and deficiency reducing angiogenesis, highlighting its regulatory role beyond extracellular matrix proteolysis [35]. The tissue inhibitor of metalloproteinases 3 has strong anti-angiogenic effects, blocking VEGF binding to vascular endothelial growth factor receptor 2 (VEGFR‑2), thereby inhibiting signaling and angiogenesis. This distinct mechanism makes TIMP-3 more effective than TIMP‑1, which primarily inhibits MMP activity [36]. Additionally, angiopoietin‑2 expands capillaries, remodels the basal lamina and drives endothelial proliferation, migration and sprouting in the presence of VEGF, yet promotes vessel regression when VEGF is absent [38]. Similarly, TNF-alpha exhibits a dual role, where low concentrations upregulate VEGF and support angiogenesis, whereas high concentrations lead to excessive inflammation and vessel regression, counteracting VEGF effects [37]. Despite these established roles, our analysis did not reveal any significant correlations between low VEGF expression levels and ICAM-I/CD54, TIMP‑1 and TIMP‑3 (Fig. 1). Furthermore, no association of angiopoietin‑2 and TNF-alpha to VEGF^low^ or VEGF^high^ could be discovered (Figs. 1 and 2). These findings suggest that VEGF regulation within ADSCs involves a more complex network beyond direct expression correlations.

Our results demonstrate that measurements of VEGF secretion are insufficient to characterize the angiogenic potential of ADSCs as there seems to be no direct correlation between this mediator and co-expression of other angiogenic adipokines. In accordance with this finding, single supplementation of VEGF may not be efficient to promote angiogenesis in the absence of contributing factors. A limitation of this study is, however, the relatively small sample size, which may restrict the strength of the conclusions drawn and the cross-sectional nature. Further factors such as hormonal influences, metabolic status and other confounding factors could not be addressed. Future studies could incorporate functional assays to validate the direct effects of these adipokines on angiogenesis and explore their interactions with other key regulators beyond VEGF, such as PDGF, transforming growth factor beta 1 (TGF-beta), and sphingolipid signaling pathways. A direct demonstration of the angiogenic capacity of ATF could be achieved through co-cultures of fat graft cells with vascular endothelial cells (ECs) which result in sprouting and formation of vascular tubes upon support by competent ADSCs [62].
